# Misconceiving patient reported outcome measures (PROMs) as primarily a reporting requirement rather than a quality improvement tool: perceptions of independent healthcare sector stakeholders in the UK

**DOI:** 10.1186/s41687-022-00511-5

**Published:** 2022-09-23

**Authors:** Michael Anderson, Emma Pitchforth, Andrew Vallance-Owen, Elias Mossialos, Paul Millner, Jon Fistein

**Affiliations:** 1 Department of Health Policy, The London School of Economics and Political Science, London, WC2A 2AE UK; 2 College of Medicine and Health, University of Exeter, Exeter, EX1 2LU UK; 3 Private Healthcare Information Network, London, W1G0AN UK; 4 Institute of Global Health Innovation, Imperial College London, London, SW7 2AZ UK; 5 School of Medicine, Cardiff University, Cardiff, CF14 4YS UK

**Keywords:** Patient reported outcome measures, PROMs, Outcome measurement, Implementation, Private healthcare, Independent healthcare, Independent providers, Theoretical domains framework

## Abstract

**Background:**

The independent healthcare sector in the UK collects PROMs for several surgical procedures, but implementation has been challenging. We aimed to understand the enablers and barriers to PROMs implementation in the independent healthcare sector in the UK.

**Method:**

Between January and May 2021, we remotely conducted semi-structured interviews with hospital consultants, hospital managers and other clinical staff using a topic guide developed from an implementation science framework called the Theoretical Domains Framework (TDF).

**Results:**

We interviewed 6 hospital consultants, 5 hospital managers, and 3 other clinical staff (1 nurse and 2 physiotherapists) across 8 hospitals. Common barriers included: the perception that PROMs are predominantly a reporting requirement rather than a quality improvement tool, absence of feedback mechanisms for PROMs data for clinicians, poor awareness of PROMs among healthcare professionals and the public, absence of direction or commitment from leadership, and limited support from hospital consultants. Common enablers included: regular feedback of PROMs data to clinicians, designating roles and responsibilities, formally embedding PROMs collection into patient pathways, and involvement of hospital consultants in developing strategies to improve PROMs uptake.

**Conclusion:**

To support PROMs implementation, independent hospitals need to develop long-term organisational strategies that involve sustained leadership commitment, goals or targets, training opportunities to staff, and regular feedback of PROMs data at clinical or governance meetings. The primary purpose of PROMs needs to be reframed to independent healthcare sector stakeholders as a quality improvement tool rather than a reporting requirement.

**Supplementary Information:**

The online version contains supplementary material available at 10.1186/s41687-022-00511-5.

## Background

Over the last few decades there have been growing efforts aimed towards implementing systems that collect and report patient reported outcomes measures (PROMs) [1, 2]. PROMs are tools or instruments, often self-completed questionnaires, to measure patient reported outcomes which are health outcomes reported directly by the patients who experience them such as quality of life, and self-reported health status [3]. While originally conceived as a research tool to facilitate the measurement of more subjective health outcomes, their potential value in clinical practice as a mechanism to measure healthcare quality and promote patient centred healthcare delivery has been increasingly recognised [4–7]. However, routine use of PROMs in clinical practice is not without challenges [8]. Barriers include a lack of resources to support implementation, complexity of PROMs, difficulty in interpreting PROMs data and professional resistance [9, 10]. While many procedure and speciality specific PROMS have been developed and implemented, response and completions rates vary significantly. An international review of published reports on PROMS with at least two follow-up time points found response rates varied from 100% to less than 30%. [11]

The UK has led efforts to promote the routine use of PROMs in clinical practice internationally with the National Health Service (NHS) in England launching a national PROMs programme in 2009 [12]. The NHS in Scotland [13], and Wales [14], have also developed national PROMs programmes launched in 2016 and 2012 respectively. Despite early enthusiasm for adopting PROMs in the NHS in the UK, the independent healthcare sector has been slower to implement PROMs in routine clinical practice. In 2014, the UK Competitions and Market Authority (CMA) published an order [15], which among other measures, mandated the collection and reporting of PROMs for privately-funded care to the Private Health Information Network (PHIN) from September 2016. In total, PHIN collects PROMs for 12 procedures across cosmetic surgery, orthopaedics, ophthalmology and urology [16]. However, the independent healthcare sector has found the implementation of PROMs challenging. Published average response rates for hip and knee replacements are much lower than in the NHS in England [17], and response rates for other procedures have not yet reached a level which warrants comprehensive publication at consultant or hospital level.

This qualitative interview study aimed to understand the enablers and barriers to improving PROMs implementation in the independent healthcare sector in the UK by undertaking semi-structured interviews with a range of stakeholders including hospital consultants, managers, nurses and physiotherapists. While there is a significant body of literature on understanding PROMs implementation in the NHS [10], to our knowledge, this is the first study which has attempted to provide insights into this particular issue in the independent healthcare sector in the UK.

## Method

### Setting

Independent healthcare providers in the UK are separate to NHS institutions and include for-profit and not-for-profit organisations delivering both NHS and privately funded healthcare services. In 2021, there were 270 independent hospitals, and 282 NHS hospitals delivering elective surgical care in England [19]. While there are similar numbers of hospitals between the two sectors, proportionally in terms of volumes the independent healthcare sector is much smaller than the NHS. In 2019, the independent healthcare sector provided approximately 1.2 million elective procedures compared to 9.7 million elective procedures delivered by the NHS hospitals [20]. Independent healthcare providers range from large national hospital groups to small specialist local providers. Most independent healthcare providers specialise in performing low complexity and high volume procedures such as cataract surgeries, hernia repair, hip replacement, and knee replacement [21]. However, there are a select number of independent hospitals that can provide a broader range of specialist services, most of which are located in London [22].

### Selection of sample

Hospital sites were selected according to several factors including: coverage of all PROMs collected by PHIN, a combination of higher and lower response rates to reflect successful as well as less successful implementation of PROMs and coverage of national independent healthcare providers. We also selected two major independent hospitals in London, as they still account for a large proportion of total volumes of elective care in the independent healthcare sector in the UK, despite not being one of the large national independent healthcare providers. We recruited participants by directly approaching hospitals to suggest relevant interviewees. Once we had interviewed one stakeholder in each hospital, if possible we used convenience sampling of known contacts to identify further interview candidates in each hospital [23]. We aimed to interview different stakeholders involved in PROMs collection or interpretation including hospital consultants, managers, nurses, and physiotherapists. The number of interviews conducted was determined by the point at which data saturation was reached. Data were analysed throughout the interview period and the point of data saturation was determined once obtaining new information became difficult [24, 25].

### Development of topic guide

To ensure a consistent approach to each interview, the co-authors of this paper developed a topic guide based upon the Theoretical Domains Framework (TDF) [26]. The TDF is an implementation science framework which is used to understand the enablers and barriers to behaviour change in healthcare professionals. It encompasses many domains including: “Knowledge”, “Skills”, “Social/Professional Role and Identity”, “Beliefs about Capabilities”, “Optimism”, “Beliefs about Consequences”, “Reinforcement”, “Intentions”, “Goals”, “Memory/Attention and Decision Processes”, “Environmental Context and Resources”, “Social Influences”, “Emotion” and “Behavioural Regulation” [26]. A full overview of the domains and constructs contained within the TDF is contained within Atkins et al. 2017 [26]. There are several other implementation science frameworks which have been developed for a similar purpose such as the Consolidated Framework for Implementation Research (CFIR) [27], and Capability Opportunity Motivation-Behavior (COM-B) model [28]. However, the TDF was chosen as it has been recommended for use with qualitative research to understand PROMS implementation specifically [29], and has been shown to produce similar findings to other implementation science frameworks such as the CFIR [30], and COM-B models. [31]

The topic guide was informed by a broader literature review of the enablers and barriers to PROMs implementation [9], and was developed iteratively amongst the co-authors. The questions included a mixture of closed and open questions. The closed questions were designed in a manner to allow quantitative analysis with positive or negative answers elicited. However, interviewees were also encouraged to elaborate on these responses to provide further insights for the qualitative analysis. The open questions were designed to encourage discussion on suggested enablers and barriers to improving PROMs implementation. The full topic guide is contained in Additional file 1: material.

### Application of topic guide

Semi-structured interviews were conducted remotely between January 2021 and May 2021 with each lasting 30 to 60 min using video conferencing software. Consistency of approach was ensured with one researcher, MA, conducting all interviews with stakeholders using the aforementioned topic guide. MA is a general practitioner with postgraduate training in health services research including training in conducting qualitative research. The interviewer did not have a prior relationship with any of the participants but built good rapport during the course of the research. However, we acknowledge that as MA is a clinician, this may have implicitly encouraged participants to consider enablers and barriers to PROMs implementations more from a clinician rather than a patient perspective. Participants were also aware that PHIN had commissioned this study. This may have improved participation as independent hospitals are encouraged to engage with PHIN as evidence of improving transparency in reporting outcomes and healthcare activity. However, this may have also influenced their answers to questions as PHIN also helps to regulate the independent healthcare sector. However, all interviewees were informed that their answers to questions were to remain anonymous. All interviews were audio recorded and notes were taken during interviews. Consent was obtained from participants both in writing and verbally beforehand. MA subsequently listened back to each interview to ensure accuracy of these notes and supplemented them with any further findings if necessary.

### Data analysis

Analysis was largely deductive in that the domains of the TDF were used as predetermined themes. Data were analysed throughout the interview period and collection was stopped when data saturation was reached (see above) [25]. Full notes from each interview were analysed using qualitative data analysis software, specifically NVivo v 1.51. Positive or negative responses to closed questions were analysed quantitatively according to hospital sites and job role and reported as percentages and absolute numbers. Whereas, further elaboration in response to closed questions and answers to open questions were summarised narratively according to each domain of the TDF. Recurring ideas were then grouped together as specific findings. These were then coded according to whether they were enablers or barriers to PROMS implementation. The decision was made that MA would lead the analysis and take sole responsibility for coding findings as it was felt this would reassure interviewees that their perspectives would remain anonymous. However, emerging findings were discussed amongst co-authors during several team meetings throughout the interview process to inform interpretations of participant responses. The results section presents summaries of the descriptive quantitative and narrative analysis.

## Results

In total, 80% (*n* = 8) of hospitals approached agreed to participate in this research. 6 hospitals were in England (2 of which were major independent hospitals in London), 1 in Wales, and 1 in Scotland. Once hospitals were approached, we were directed towards the chair of the Medical Advisory Committee (MAC) in 75% (*n* = 6) of hospitals to be interviewed. The remaining 25% (*n* = 2) of hospitals directed us towards a hospital manager to be interviewed. MAC Chairs are working hospital consultants that have been granted earmarked time by the independent hospital to fulfil additional managerial responsibilities. 63% of hospitals (*n* = 5) suggested one or more additional stakeholders to be interviewed. 55% (*n* = 6) of the additional stakeholders that were suggested subsequently agreed to be interviewed. In total, fourteen stakeholders were interviewed including 6 hospital consultants, 5 hospital managers, and 3 other clinical staff (1 nurse and 2 physiotherapists). All hospital consultants were male and at least 10 years post completion of specialty training. 60% (*n* = 3) of hospital managers were female and 100% (*n* = 5) hospital managers held senior management positions in their respective organisations. 67% (*n* = 2) of other clinical staff were female and all had at least 5 years’ experience post qualification.

Table 1 offers aggregate summaries of the enablers and barriers to PROMs implementation identified during interviews classified according to the aforementioned TDF. Following this there is a narrative account of the thematic analysis of findings that follows the domains.

**Table 1:** Enablers and barriers discussed during interviews

Domain	Enablers	Barriers
Knowledge	Regular communication on value of patient reported outcome measures (PROMs) from hospital-level leadership, corporate-level leadership and the private health information network (PHIN)*	Poor awareness that collection and reporting of PROMs is mandated by the Competition and Markets Authority (CMA) Poor awareness that collection of PROMs extends beyond knee and hip replacement
Skills	Training opportunities on skills required for collection, submission or interpretation of PROMs	Limited skills for hospital managers in interpreting PROMs data
Social/ professional role and identity	Designating responsibility for the PROMs collection and submission process to an individual or individuals, ie pre-operative administrators or clinical nurse specialists	Limited or no involvement of hospital consultants in development of processes for collection, submission and reviewing of PROMs* Not recognized by some hospital consultants that it was their responsibility to encourage the use of PROMs
Belief about capabilities	Embedding PROMs into patient pathways through policies and procedures Incorporating PROMs within pre-operative assessment documentation Designated drop-off points for completed PROMs forms	Absence of dedicated personnel or processes in place for PROMs collection* Absence of forum to review and act on PROMs data*
Optimism	Adequate volumes of procedures Benchmarking performance against other hospital consultants Use of validated PROMs instruments Appropriate case-mix adjustment when reporting PROMs data*	Absence of clear indication for certain PROMs instruments Perception of inconclusive or mixed evidence regarding value of specific PROMs instruments Absence of feedback mechanisms for PROMs data*
Belief about Consequences	Regular feedback and discussion of PROMs data at clinical meetings* Greater role for insurers in accessing and reviewing PROMs data to guide patient decisions	Perception that PROMs are a reporting requirement rather than beneficial for quality improvement Absence of feedback mechanisms for PROMs data* Poor public awareness about PROMs
Reinforcement	Routine benchmarking of hospital performance in terms of PROMs response rates Regular reminders through newsletters, meetings, or information boards on wards CMA and/or PHIN holding hospitals accountable that do not collect or report PROMs	Poor awareness among hospital consultants of reinforcement mechanisms for PROMs
Intentions	Developing a corporate-level or hospital-level strategy to improve PROMs uptake Involvement of hospital consultants in development of strategies to improve response rates*	Absence of direction from corporate level leadership regarding value and importance of PROMs*
Goals	Setting hospital-level targets for PROMs participation and completion rates*	Setting a target in isolation without actions to improve awareness, train staff and feedback data
Memory, attention and decision processes	Ensuring staff have the right tools to aid communication with patients such as patient information leaflets Allowing patients to choose whether to complete PROMs forms in outpatient clinics or later via post or electronically	Patients experiencing “form-fatigue” when overwhelmed with forms to complete in outpatient clinics Complexity of processes involved in identifying eligible patients, data protection and submitting data
Environmental context and resources	Designing patient pathways whereby the collection of PROMs becomes a by-product of care Emphasising the value of PROMs to demonstrate quality of care as a hospital marketing strategy	Limited capacity within governance teams to monitor compliance with processes involved in collection, submission or reviewing of PROMs
Social influences	Commitment from hospital and corporate level leadership to improve PROMs uptake* Developing a long-term strategy to improve PROMs uptake rather than “one-off” initiatives Avoiding a blame culture for hospital consultants with lower than average PROMs scores*	Limited support for PROMs from hospital consultants, particularly if perceived as solely a monitoring exercise Different approaches to PROMs in the NHS across the four countries of the UK
Emotion	Confidence that data is not misleading through appropriate case-mix adjustment, and caveats that acknowledge limitations in data Reporting at hospital-site level rather than individual hospital consultant level	Misleading data if not representative of breadth of practice across NHS and independent health sector or complexity of patients
Behavioural Regulation	Reviewing PROMs data at clinical or governance meetings* Greater emphasis on PROMs within hospital consultant appraisal and revalidation	Absence of awareness among hospital consultants of mechanisms to monitor compliance with processes involved in PROMs

^*^These are bi-directional with the converse statement discussed as either a barrier or enabler

### Knowledge

All stakeholders (100%, *n* = 14) interviewed were aware of the use of PROMs in the independent healthcare sector, however there was mixed awareness that hospitals were mandated by the CMA order to report on PROMs among hospital consultants (50%, *n* = 3), hospital managers (80%, *n* = 4), and clinical staff (67%, *n* = 2). Most stakeholders understood PROMs as a strategy to monitor and improve quality of care, but PROMs were thought to be more applicable to orthopaedic surgery than any other specialties. It was commented during interviews that the UK devolved administrations in Wales and Scotland do not routinely utilize PROMs in the NHS to the same extent as in England and this inevitably has some spill-over effect in awareness in the independent healthcare sector. Suggested enablers to improving awareness included improved and regular communication on the rationale and value of using PROMs. Hospital consultants and other clinical staff generally felt this communication needed to come from hospital level and corporate level management, whereas 40% of hospital managers (*n* = 2) felt there was an unmet need for improved communication from PHIN.

### Skills

All hospital consultants (100%, *n* = 6) interviewed felt they had the skills to use and interpret PROMs data, stating they are routinely expected to do this either when reviewing academic research or as part of their NHS work. Similarly, all other clinical staff (100%, *n* = 3) also felt equipped with the relevant skills required to support the process of PROMs collection. However, some hospital managers (40%, *n* = 2) did not feel they have the necessary skills to interpret PROMs data and felt this would need to be addressed if reviewing PROMs data for individual hospital consultants was to become more routine. Suggested enablers included regular training for staff, with stakeholders from 25% of hospitals (*n* = 2) explaining how regular training led by a clinical nurse specialist or physiotherapist on the skills required for the collection and submission of PROMs data had helped improve response rates.

### Social/professional role and identity

There were mixed perceptions from hospital consultants regarding their role in encouraging the use of PROMs. 33% of hospital consultants (*n* = 2) interviewed felt this was not their responsibility, whereas the remaining 67% of hospital consultants (*n* = 4) felt they had a responsibility to encourage the use of PROMs within the wider multidisciplinary team through communication with patients and regular discussion during clinical and governance meetings. There was more consensus among hospital consultants that their role involved using PROMs data to monitor and improve quality of care but they emphasised that PROMs data feedback rarely happens. 20% of hospital managers (*n* = 1) felt that hospital consultants were too detached from development of processes for the collection, submission and reviewing of PROMs and by not involving them in this process, this reduced their engagement. All hospital managers (100%, *n* = 5) felt their role involved monitoring compliance against the processes involved in the collection and reporting of PROMs data, however only 60% of hospital managers (*n* = 3) felt their role involved feeding back PROMs data at clinical or governance meetings. All other clinical staff (100%, *n* = 3) interviewed felt there was clarity in their role to support the collection of PROMs through identifying eligible patients and communication with patients to explain the rationale and processes involved. There was consensus among interviewed stakeholders that designating responsibility to an individual or individuals for PROMs collection within the pre-operative clinic was a strong enabler to improve response rates.

### Belief about capabilities

The majority of hospital consultants (83%, *n* = 5), hospital managers (60%, *n* = 3) and other clinical staff (67%, *n* = 2) felt confident in their capabilities to support the collection, submission and interpretation of PROMs. 13% of hospital consultants (*n* = 1) interviewed did not feel confident as there was no dedicated personnel or processes in place for PROMs collection and instead they were collected on an ad-hoc basis. 40% of hospital managers (*n* = 2) interviewed did not feel confident in their capabilities as they felt there was no forum to review and act on PROMs data since they were not routinely discussed during clinical or governance meetings. Suggested enablers to improve capabilities for collection included formally embedding PROMs into patient pathways through policies and procedures, incorporating PROMs forms within pre-operative assessment documentation and ensuring there was clarity regarding designated drop-off points for completed PROMs forms.

### Optimism

All hospital consultants (100%, *n* = 6) interviewed agreed that the use of PROMs data has significant potential to improve quality of care. However, they felt this was dependent upon several factors such as adequate volumes of procedures undertaken by hospitals or hospital consultants, benchmarking against other hospital consultants, the use of validated PROMs instruments, appropriate case-mix adjustment and regular feedback to hospital consultants. Barriers included absence of clear indication on when to use certain PROMs instruments, inconclusive or mixed evidence regarding value of specific PROMs instruments and absence of feedback mechanisms for PROMs data. The opinion of other healthcare professionals was mixed with 67% of other clinical staff (*n* = 2) and 20% of hospital managers (*n* = 1) stating they were not convinced PROMs improved quality of care and needed further rationale or evidence to change their perspective.

### Belief about consequences

There was consensus among hospitals that PROMs data are rarely used for local quality improvement purposes in private hospitals. Stakeholders from only 40% of hospitals (*n* = 2) discussed PROMs data being analysed for individual hospital consultants. However, reviewing hospital response rates for PROMs was more commonplace with 100% of hospital managers (*n* = 5) and 67% of other clinical staff (*n* = 2) stating these were reviewed during governance meetings. However, hospital consultants were less aware of this with only 33% of hospital consultants (*n* = 2) aware of the response rates for their hospital. Barriers included a perception among stakeholders that the collection and submission of PROMs data was to comply with a reporting requirement outlined by the CMA and PHIN rather than for quality improvement, an absence of feedback mechanisms for PROMs data and poor interest or prioritization regarding PROMs data at governance meetings. Stakeholders from 13% of hospitals (*n* = 1) discussed how the recent introduction of regular feedback of PROMs data at governance meetings and within newsletters had been responsible for significant increases in response rates for PROMs.

83% of hospital consultants (*n* = 5), 40% of hospital managers (*n* = 2), and 67% of other clinical staff (*n* = 2) felt that patients were not currently using PROMs data reported nationally to compare the performance of healthcare providers. Instead, they felt that patients were more influenced by factors such as geography, family or friend recommendation, GP referral or healthcare comparison websites. However, it had been emphasised that PHIN has only published PROMs data for less than two years. Barriers discussed included poor awareness among the public of the existence of PHIN, and how to interpret PROMs data. One suggested enabler discussed was facilitating a greater role for insurers in accessing and reviewing PROMs data to guide patient decisions in accessing healthcare providers.

### Reinforcement

There were mixed findings in terms of reinforcement mechanisms to encourage the use of PROMs. 83% of hospital consultants (*n* = 5) interviewed stated there were not aware of any mechanism to reinforce the use of PROMs, whereas 100% of hospital managers (*n* = 5) and all other clinical staff (*n* = 3) were aware of some mechanisms for this purpose such as incorporating PROMs forms within pre-assessment documentation or protocols, reviewing response rates through information boards and regular reminders through either hospital or corporate level newsletters, clinical meetings, or governance meetings. Other suggested enablers included the CMA holding hospitals accountable that do not collect or report PROMs or routine feedback and benchmarking in terms of PROMs response rates from either PHIN and/or the head office of each respective healthcare provider.

### Intentions

67% of hospital consultants (*n* = 4) interviewed stated they had no intentions to improve uptake of PROMs in their hospitals, with the remaining 33% (n = 2) stating that engaging with this research had made them reconsider their hospital’s approach to PROMs and now had plans to explore ideas and opportunities to improve uptake of PROMs. 40% of hospital managers (*n* = 2) and 67% of other clinical staff (*n* = 2) stated they had intentions to improve uptake as PROMs as their hospitals were mid-way through initiatives to improve their response rates for PROMs. Suggested enablers to encourage hospitals to make plans to improve uptake of PROMs included developing a company-wide or hospital-level strategy to improve PROMs uptake, involvement of hospital consultants in the development of strategies to improve response rates, regular review and feedback of response rates to managerial and clinic staff through governance meetings and newsletters, and enhanced training on rationale and process of PROMs. Whereas, a barrier discussed was absence of direction from corporate level leadership regarding value and importance of PROMs.

### Goals

Stakeholders from only 13% of hospitals (*n* = 1) were aware of a target for improving PROMs participation rates. Specifically, they were aiming to achieve 90% participation for pre-operative PROMs in orthopaedic surgery, which they cited as a strong enabler. When questioned, there was consensus among hospital stakeholders, including 83% of hospital consultants (*n* = 5), 100% of hospital managers (*n* = 5) and 100% of other clinical staff (*n* = 3) that setting an organizational target or goal could be a useful enabler to improve uptake of PROMs in the future. However, 20% of hospital managers (*n* = 1) were keen to emphasise that setting a target alone would not be a sustainable strategy to improve response rates as this can encourage gaming of the system. It was thought that this must be combined with other actions to improve awareness of PROMs, train staff and facilitate regular feedback of data.

### Memory, attention and decision processes

83% of hospital consultants (*n* = 5) did not think that the processes involved in PROMs were complex or difficult to engage with, stating again that they were familiar with PROMs due to exposure to PROMs when reviewing academic research or as part of their NHS work. Similarly, 100% of other clinical staff (*n* = 3) did not think the processes involved in PROMs collection were complex, although they commented how patients do sometimes find it challenging to understand rationale for PROMs and often experience “form-fatigue” when filling in large amounts of paperwork at outpatient clinics. 33% of other clinical staff (*n* = 1) felt asking patients to fill in PROMs questionnaires in outpatient clinics may not be the ideal setting as some patients may feel pressured or nervous and that creating mechanisms for patient choice to complete forms by post or electronically may be one way to overcome this. Another enabler discussed was ensuring staff had the right tools to aid communication with patients such as patient information leaflets that explain rationale of PROMs. There were, however, different perspectives from hospital managers with 80% of hospital managers (*n* = 4) stating the processes involved in identifying eligible patients, ensuring compliance with data protection processes and formatting and submitting data to PHIN meant that collecting and submitting PROMs was a time consuming and complex process.

### Environmental context and resources

83% of hospital consultants (*n* = 5) felt that they had enough time to engage with the processes involved in PROMs, however the remaining 13% of hospital consultants (*n* = 1) felt it was challenging to encourage hospital consultants to engage with PROMs if using additional time in a sector where each unit of hospital consultant time or patient seen had clear financial consequences. 67% of other clinical staff (*n* = 2) felt they had enough time to engage with the processes involved in PROMs. The remaining 33% of other clinical staff (*n* = 1) stated that they felt they did not have adequate time but nonetheless found time to engage as it had become a compulsory aspect of their job. There was consensus among all hospital consultants (100%, *n* = 6) and all other clinical staff (100%, *n* = 3) that independent sector hospitals should have enough resources to support the use of PROMs. Hospital managers expressed different perspectives with 80% (*n* = 4) stating they had limited time or resources to engage with the processes involved in monitoring compliance with PROMs, with a key barrier being limited capacity in governance teams which often have multiple and increasing responsibilities. A suggested enabler to ensuring there was adequate time to facilitate the collection of PROMs was designing pathways whereby the collection of PROMs data becomes a by-product of delivery care. A suggested enabler to securing necessary resources included emphasising the value in using PROMs to demonstrate the quality of care to potential patients as a hospital marketing strategy.

### Social influences

There were mixed findings in terms of social influences reported from hospital consultants. 67% of hospital consultants (*n* = 4) felt their colleagues did not support the use of PROMs, whereas the remaining 33% of hospitals consultants (*n* = 2) felt they did. This was in contrast to 100% of hospital managers (*n* = 5) and 67% of other clinical staff (*n* = 3) that felt their hospital consultant colleagues did not always support the use of PROMs, although they felt the younger generation of hospital consultants were more supportive of PROMs. It was repeatedly discussed how hospital consultants often perceive PROMs as more of a monitoring exercise rather than an opportunity to improve quality and patient centredness of care. It was again mentioned that the NHS in the four UK countries have different approaches to PROMs that subsequently has spill-over effects in terms of hospital consultant engagement within the independent healthcare sector. Suggested enablers included commitment from hospital and corporate level leadership to improving PROMs uptake, developing a long-term organisational strategy to improve engagement with PROMs rather than one-off initiatives and avoiding a blame culture for hospital consultants with lower than average PROMs scores.

### Emotion

There were also mixed findings for concerns regarding the use and reporting of PROMs from hospital consultants with 50% (*n* = 3) stating they had no concerns and 50% (*n* = 3) stating they did. Concerns raised included that data could be misleading if incomplete or did not represent the breadth of their practice across both the NHS and private healthcare sector. Another concern raised was that data may not be representative if some hospital consultants took on more complex patients and that publication of PROMs data may even encourage case selection. All hospital managers (100%, *n* = 5) and all other clinical staff (100%, *n* = 3) did not express any concerns regarding the use and reporting of PROMs but acknowledged their hospital consultant colleagues had voiced such concerns during clinical or governance meetings. Suggested enablers to improve confidence in reported data included appropriate case mix adjustment and caveats when reporting data that acknowledges any limitations in interpretation. Other enablers included reporting at hospital-site level rather than hospital consultant level to avoid any individual hospital consultant being unfairly penalised for inaccurate data.

### Behavioural regulation

All hospital managers (100%, *n* = 5) were aware of mechanisms to monitor compliance with the processes involved with PROMs with the most discussed mechanism being feedback of response rates from system suppliers who are contracted to collate, analyse and submit PROMs data. This data would then be reviewed subsequently at clinical or governance meetings. There was lower awareness among hospital consultants and other clinical staff, with only 40% of hospital consultants (*n* = 2) and 33% of other clinical staff (*n* = 1) aware of such processes. Another enabler to improving uptake of PROMs when discussed by hospital consultants was including a greater emphasis on PROMs within their regular appraisal and revalidation activities. However, 13% of hospital consultants (*n* = 1) expressed concern that PROMs were adding an additional administrative burden to an already crowded regulatory environment related to their practice.

## Discussion

### Summary of findings

This study has identified several enablers and barriers that influence PROMs implementation in the independent healthcare sector in the UK. While there was broad consensus among interviewees regarding the potential added value of using of PROMs, they discussed how PROMs are predominantly perceived as a reporting requirement and rarely used at the local level for quality improvement purposes in practice. A re-occurring finding across domains was an absence of feedback mechanisms for PROMs data, which drives this misperception. Other commonly discussed barriers included poor awareness of PROMs among healthcare professionals and the public, absence of direction or commitment from corporate level leadership and limited support from hospital consultants. Many barriers and enablers were bi-directional and regular feedback of PROMs data (either through clinical or governance meetings, in newsletters or information boards in wards) were repeatedly discussed as a strategy for positive reinforcement. Other enablers included designating responsibility for PROMs collection and submission to an individual or individuals in the pre-operative clinic, formally embedding PROMs collection into patient pathways through policies or procedures and involvement of hospital consultants in developing strategies to improve PROMs uptake. Hospital consultants expressed concerns that reported PROMs data could be misleading if not representative of practice breadth or complexity of patients. Strategies to overcome these fears including ensuring adequate volumes of procedures before reporting, appropriate case-mix adjustment and reporting data at hospital level rather than individual hospital consultant level. It was also stressed during interviews that a multicomponent and long-term strategy is required for a sustainable approach to improving PROMs implementation that incorporates sustained commitment from hospital and corporate level leadership, settings goals or targets, training opportunities for staff, regular feedback of PROMs data at clinical or governance meetings, and avoiding a blame culture.

### Comparison with previous literature

This study shares many findings with previous literature that has explored enablers and barriers to PROMs implementation. A realistic review into PROMs implementation emphasised that if there is a perception that PROMs are being imposed by an external agency this may encourage gaming of the system and lack of engagement, with this being a major barrier for implementation [10]. As we find that many independent healthcare sector stakeholders feel that PROMs are a mandatory reporting requirement imposed upon them, this may explain why limited support from hospital consultants was described by participants. Similar to our study, poor awareness among healthcare professionals and patients about PROMs and their objectives is a frequently cited barrier in previous literature [9, 10, 32–35]. This study emphasises enablers to overcome this barrier similar to previous literature such as training opportunities provided for staff, [9, 33, 36, 37] regular communication and feedback of data [9, 10, 32–40] and the designation of clear roles and responsibilities in relation to PROMs collection and interpretation. [9, 32, 39–41] However, there are several other enablers described in the literature that are not discussed in this study, such as providing opportunities to trial the use of PROMs before more widespread implementation [9, 39, 41, 42], the involvement of patients or their representatives in designing pathways or protocols for PROMs implementation [9, 39, 41, 42] and the availability of sufficient statistical support to appropriately analyse and interpret PROMs data. [10, 32, 33] However, the conclusion of this study, that successful PROMs implementation is dependent upon investing time and resources into designing and sustaining multicomponent PROMs strategies at the hospital level, is shared among many other reviews of PROMs implementation studies. [9, 34, 35]

### Strengths and limitations

To our knowledge, this study is the first study which aims to understand the barriers and enablers to PROMs implementation in the independent healthcare sector in the UK. This is a major strength of our paper as it is more challenging to conduct research in independent sector hospitals in the UK than in NHS hospitals which often have an academic affiliation or a local research and development department.

However, there are some limitations to our analysis which need to be considered. First, there are potential sources of selection bias in our sample. All hospital consultants interviewed were MAC chairs, which have earmarked time for managerial responsibilities reimbursed by their respective hospitals. Therefore, it is possible their perspectives may not reflect the perspectives of the average hospital consultant practicing in the independent healthcare sector. We openly acknowledge this possibility, however all MAC chairs interviewed remain practicing hospital consultants and therefore we believe their perspectives remain relevant. Moreover, we used convenience sampling of known contacts and there is a risk that this approach may have directed us towards stakeholders which share common perspectives and opinions, restricting the potential for collecting a broader range of information from interviews. However, convenience sampling is often used in exploratory work in areas of new research and particularly in fields where it would be challenging to recruit participants in alternative ways [43]. We argue this is the case in the independent healthcare sector in the UK as hospital consultants typically work on a fee for service basis. Therefore, it is more challenging to secure engagement with research on an unpaid basis than within the NHS where hospital consultants are usually reimbursed by salaried contracts. Moreover, it was not feasible to conduct random sampling of healthcare professionals as there is no common database of healthcare staff across the independent healthcare sector available.

### Recommendations for practice and research

To make further progress in strengthening PROMs implementation, independent hospitals need to develop long-term strategies to support this. Several priorities for inclusion have been identified by this study including committed leadership, a supportive culture, setting targets, designated roles and responsibilities, regular feedback of PROMs data and greater engagement of hospital consultants. While strengthened regulation of PROMs collection by the CMA through mechanisms such as fines or penalties for non-compliant independent hospitals is one option, previous literature has emphasised that the perception that PROMs collection are being enforced by an external agency can be a major barrier to implementation and engagement at the local level [9]. Instead, sustainable implementation will be dependent upon convincing independent healthcare sector stakeholders of the added value of routinely using PROMs to measure healthcare quality and promote patient centred healthcare delivery.

While there are strengths to our study, we have also identified limitations which could be addressed in further research which builds on this study. We hope this study will increase interest in this area from researchers and funders. With more resources, further research could incorporate a broader sample with a greater variety of participants in relation to their disciplines and career experience. With improved data collection on the independent healthcare sector workforce, there may also be opportunities to utilise surveys to gain insights from larger samples and apply random sampling techniques to improve the representativeness of samples. There is also an unmet need for operational research which details which patient pathways, communication tools and health information technology infrastructure are needed to help improve PROMs collection.

## Conclusions

Following interviews with a variety of stakeholders across the independent healthcare sector in the UK, we have found that PROMs are predominantly perceived as a reporting requirement rather than a quality improvement tool. This misperception is being driven by an absence of feedback mechanisms, poor awareness of PROMs and limited support from corporate level leadership and hospital consultants. The development of hospital-level multicomponent and long-term strategies that encompass sustained leadership commitment, settings goals or targets, training opportunities and regular feedback of PROMs data will be needed to improve PROMs implementation.

Considering the significant efforts that have taken place to understand PROMs implementation in NHS hospitals, it is notable that this is the first study that has focused on this issue in the independent healthcare sector in the UK. There is a need for further research to build upon this study and monitor implementation of the aforementioned strategies. However, this will require improved funding, earmarked time for healthcare professionals to participate, partnerships with universities and academic departments, and engagement from independent hospitals to interpret and implement findings.

## Supplementary Information

**Additional file: 1 **PHIN PROM project qualitative research topic guide

## Data Availability

The topic guide used for this qualitative research is available in Additional file 1.
