# Supplementary material for: Misconceiving patient reported outcome measures (PROMs) as primarily a reporting requirement rather than a quality improvement tool: perceptions of independent healthcare sector stakeholders in the UK
Source: J Patient Rep Outcomes. 2022 Sep 23;6:101. doi: 10.1186/s41687-022-00511-5 (PMC9500124; doi:10.1186/s41687-022-00511-5)
Supplement: Supplementary file 1 — Additional file: 1 PHIN PROM project qualitative research topic guide [file 41687_2022_511_MOESM1_ESM.docx]

**Supplementary Material: PHIN PROM Project Qualitative Research Topic Guide**

The below topic guide has been developed using the Theoretical Domains Framework (TDF) (Table 1),^1^ a commonly used framework in implementation science to understand the enablers and barriers for successful implementation of quality improvement strategies and sustainable behaviour change of healthcare professionals.

**Knowledge**

1. What do you know about patient report outcome measures (PROMs) in the context of the independent healthcare sector in the UK?
2. Are you aware that all consultants and hospitals in the independent healthcare sector are mandated by the Competition and Markets Authority to collect PROMs for certain procedures?
3. What do you think could be done to improve awareness of PROMs in the independent healthcare sector in the UK?

**Skills**

1. Do you think you or your colleagues in your hospital have the relevant skills to encourage the use of PROMs?
2. What do you think could be done to ensure you or your colleagues are equipped with the necessary skills to encourage the use of PROMs?

**Social/professional role and identity**

1. How do you see your role in supporting the use of PROMs?
2. How could your role be utilised more effectively to support the use of PROMs?

**Belief about capabilities**

1. How confident are you in your capability to support the processes involved in the collection, submission, or interpretation or PROMs?
2. If not, what steps do you think could be taken to improve your capability to support the processes involved in the collection, submission, or interpretation of PROMs?

**Optimism**

1. Do you think that that the use of PROMs has the potential to improve the quality and patient centredness of care?
2. If not, what makes you sceptical regarding their potential benefit?

**Belief about Consequences**

1. Do you believe that PROMs are used effectively within your hospital to improve quality and patient centeredness of care?
2. Do you believe PROMs data collected and reported nationally are used by patients and other stakeholders to effectively compare the performance of healthcare providers?
3. What steps do you think could be taken at either the hospital or national level to ensure PROMs are used effectively?

**Reinforcement**

1. What mechanisms exist to encourage or reinforce the use of PROMs in your hospital?
2. Are these mechanisms effective, and if not, what alternative mechanisms would you suggest?

**Intentions**

1. Do you have any plans to improve uptake of PROMs in your hospital?
2. If not, what would make you change your position and consider efforts to improve uptake of PROMs?
3. Are there any other individuals in your hospital who have expressed an interest in improving uptake of PROMs?

**Goals**

1. Does your hospital or the Medical Advisory Committee have a clear target or goal set for improving uptake of PROMs?
2. If not, do you believe setting a goal or target to improve uptake of PROMs in your hospital would be a helpful enabler to improving uptake?

**Memory, attention and decision processes**

1. Have you or your colleagues found that the use of PROMs is complex or difficult to engage with?
2. If so, what steps would you recommend to simplify the processes involved in using PROMs?

**Environmental context and resources**

1. Do you or your colleagues have enough time available to support the use of PROMs?
2. Do you think your hospital has enough resources to support the use of PROMs ?
3. If not, what steps could be taken to secure the necessary resources and/or time to support the use of PROMs in your hospital?

**Social influences**

1. Do you feel your colleagues support the use of PROMs to improve quality and patient centredness of care?
2. If not, what steps could be taken to change the culture within your hospital to achieve this goal?

**Emotion**

1. Do you or your colleagues have any concerns about the use and/or reporting of PROMs?
2. If so, what steps do you think could be taken to address these concerns?

**Behavioural Regulation**

1. Are there any people or mechanisms in place which monitor compliance with processes involved in using PROMs in your hospital?
2. If not, what mechanisms or persons do you think should be involved in the monitoring compliance with processes involved in using PROMs in your hospital?
